# The Growth of the Nursing Profession: III. The Work of Reorganisation

**Published:** 1918-02-09

**Authors:** 


### THE MATRONS' AND SISTERS' DEPARTMENT.

### THE GROWTH OF THE NURSING PROFESSION.

### III. The Work of Reorganisation.

WE have seen that the College of Nursing, with its Council elected by the nurses on the College Register, will be in a position to carry out the work for which a representative body was needed. This work is nothing less than the reorganisation of the nursing profession, with due regard to an equilibrium of interests. Now, in all professions and trades which have pursued their course for any length of time without the guidance of a central authority, different persons connected with them acquire certain claims upon which time sets its seal with the title of "vested interests." There is, perhaps, no more effectual bar to clear thinking and straightforward progress than these same vested interests. They cannot be ignored, for this would involve injustice. But while they make the task of reorganisation extremely difficult and complicated, they must not be allowed to assume such dimensions as to obscure our conception of our central idea and its realisation.

All professions have a twofold basis, economic and educational, and neither must be neglected in favour of the other. Especially is this so in the nursing profession, where the economic basis has hitherto been so entirely neglected that it was apparently non-existent. This failure to recognise the necessity for a sound economic basis has reacted to its detriment upon the educational aspect of the profession, and it will be useless to draw up attractive plans for one wing of the building unless at the same time provision is made for a solid foundation for the other.

Keeping in mind this twofold basis, let us try to obtain a clear formulation of the objects to be held in view in undertaking the work of reorganisation. There are three several standpoints to be taken into consideration. Perhaps the most important, as affecting the largest number of persons, is that of the community for whose benefit the profession exists. What are the ideal requirements of the community as far as nursing is concerned? In the first place, promptitude of service for all classes and in all emergencies, so that not one person need suffer for the want of immediate attention. In the second place, wise and economical expenditure of all moneys granted by the State as representing the community, or by the generosity of individual donors. In the third place, the highest standard of individual efficiency as regards character, education, and professional training, so that by no possible chance can the profession be reduced to a soulless husk of mechanical organisation. In a word, what the community requires is co-ordinate efficiency.

Then there is the professional standpoint. Any body of persons following the same calling and having more or less the same interests must necessarily be conscious of a corporate life. The first object of this corporate life in the case of nursing is service of the community. Service, but not slavery. Service ennobles, slavery degrades. It may seem absurd in the twentieth century to recall that fact, but none the less the lives of many groups of nurses in this year of grace 1918 are not far removed from slavery. They are, at all events, "cribb'd, cabin'd and confined." The profession requires public recognition as one of the highest callings which minister to the community, and with that recognition an independence which will enable it to attend to the growing needs, and to give room for the ever-increasing development in all directions which mark the growth of corporate, as of individual life. Thus, put shortly, the object in view from the professional standpoint is independence and breadth of scope.

Then lastly, but by no means to be ignored, is the individual standpoint. Neither man nor woman lightly chooses a profession without first considering what scope it offers for the development of powers as yet, perhaps, existent only in embryo, but none the less quite consciously existent, and demanding space wherein to grow and flourish and attain maturity. The self-repression which characterised the ideal of woman in an earlier era is not now manifested, as it was then, in an uncomplaining acceptance of narrow horizons. The majority of the best nurses will always be inspired primarily by the pure desire to be of service to the sick, but this desire will be accompanied by the determination to attain the largest possible mental and spiritual growth, and the complete unfoldment of every latent power the woman possesses, and she will therefore demand that the conditions of her service shall give her opportunities for the full and free development of her faculties. This individual standpoint is one which it is very necessary to keep in view, so that, in putting its hand to the task of reorganisation, the College may have a clear conception of the needs of all those who are going to be affected by such reorganisation.

